# Bioequivalence of Esaxerenone Conventional Tablet and Orally Disintegrating Tablet: Two Single‐Dose Crossover Studies in Healthy Japanese Men

**DOI:** 10.1002/cpdd.1087

**Published:** 2022-03-21

**Authors:** Akifumi Kurata, Takashi Eto, Junko Tsutsumi, Yoshiyuki Igawa, Yasuhiro Nishikawa, Hitoshi Ishizuka

**Affiliations:** ^1^ Quantitative Clinical Pharmacology Department Daiichi Sankyo Co., Ltd. Tokyo Japan; ^2^ Souseikai Hakata Clinic Fukuoka Japan; ^3^ Data Intelligence Department Daiichi Sankyo Co., Ltd. Tokyo Japan; ^4^ Development Strategy & Planning Group Daiichi Sankyo Co., Ltd. Tokyo Japan

**Keywords:** bioequivalence, esaxerenone (CS‐3150), hypertension, mineralocorticoid receptor blocker, orally disintegrating tablets

## Abstract

We assessed the bioequivalence of a single dose of 5‐mg of esaxerenone administered as an orally disintegrating tablet (ODT) with the conventional oral tablet in healthy Japanese men. This single‐center, open‐label, randomized, two‐drug, two‐stage crossover, single‐dose study was conducted in two parts. In study 1, both formulations were taken with water. In study 2, only the ODT formulation was taken without water. The primary outcome was the evaluation of bioequivalence of the ODT and conventional tablet using the pharmacokinetic (PK) parameters maximum plasma concentration (C_max_) and area under the plasma concentration–time curve to the last quantifiable time (AUC_last_). Plasma concentrations were measured using a validated liquid chromatography/mass spectrometry method and PK parameters were calculated by noncompartmental analysis. The ratios of the geometric least‐squares mean (2‐sided 90% confidence intervals [90%CIs]) for ODT with (study 1) and without (study 2) water to the conventional tablet were 1.03 (1.00–1.07) and 1.01 (0.96–1.06) for C_max_ and 1.03 (1.00–1.07) and 0.96 (0.94–0.98) for AUC_last_, respectively. The 90%CIs fell within the predefined bioequivalence range of 0.80–1.25. Treatment‐emergent adverse events were similar between both formulations. In conclusion, esaxerenone 5‐mg ODT taken with or without water was bioequivalent to a single 5‐mg conventional oral tablet.

Hypertension is a well‐recognized public health problem in Japan, and it has been estimated that approximately 43 million Japanese individuals have some form of the condition.^1^ This equates to approximately one‐third of the overall population and is due primarily to Japan's aging society. As hypertension is the single most important risk for cerebrocardiovascular events,^2^ adequate treatment is extremely important from both the clinical and socioeconomic viewpoints.

Despite treatment, many Japanese patients with hypertension have poor blood pressure control.^3^ Many patients are elderly (65 years or older) and may have comorbidities or health issues that decrease antihypertensive treatment adherence. In particular, dysphagia may make it difficult to comply with the requirement to take conventional oral tablets.^4^

Orally disintegrating tablets (ODTs) are now widely used across multiple treatment indications and have several benefits over conventional tablets. ODTs are clinically attractive because they are suitable for use in patients with dysphagia, improve adherence and increase the likelihood of achieving the desired therapeutic effect, reduce the pill burden for patients who require multiple medications, and are convenient to take without water.^5^, ^6^

Esaxerenone is an oral, nonsteroidal, selective mineralocorticoid receptor blocker,^7^, ^8^, ^9^, ^10^ which was approved in Japan in January 2019 for the treatment of hypertension.^11^ Esaxerenone has demonstrated antihypertensive activity in a variety of patients, including those with uncomplicated grade I–III hypertension,^12^, ^13^, ^14^ hypertension with moderate renal impairment,^15^ hypertension with type 2 diabetes mellitus and albuminuria,^16^, ^17^, ^18^ and hypertension associated with primary aldosteronism.^19^

Esaxerenone is eliminated via multiple pathways and several metabolites are formed by cytochrome P450 (CYP) 3A, 5′‐diphospho‐glucuronosyltransferase (UGT), and hydrolysis followed by glucuronidation, with CYP3A‐mediated oxidation contributing to almost one‐third of esaxerenone clearance.^20^ The potential for drug–drug interactions (DDIs) was observed when esaxerenone exhibited time‐dependent weak inhibition then induction of CYP3A activity in vitro.^21^ In healthy volunteers, itraconazole, a strong inhibitor of CYP3A, and rifampicin, a strong inducer of CYP3A, increased and reduced, respectively, the esaxerenone area under the plasma concentration–time curve (AUC) from 0 to infinity (AUC_0‐∞_),^22^ suggesting caution is required when coadministering esaxerenone with strong inhibitors and inducers of CYP3A. However, based on the lack of clinically relevant pharmacokinetic (PK) changes in healthy volunteers, the risks of DDI are expected to be low during combination therapy with esaxerenone and amlodipine,^23^ a substrate and weak inhibitor of CYP3A, digoxin,^23^ a P‐glycoprotein (P‐gp) substrate, or midazolam,^24^ a sensitive CYP3A substrate.

Among antihypertensive treatments, ODTs are available or in development for several drug classes.^25^, ^26^, ^27^, ^28^, ^29^, ^30^ To date, no ODT formulations have been available for any mineralocorticoid receptor blockers; however, a new ODT formulation of esaxerenone is now in development for the purpose of improving convenience and compliance for patients with hypertension. This study was designed to assess the bioequivalence of a single, 5‐mg dose of esaxerenone administered as an ODT with the conventional oral tablet in healthy Japanese male subjects.

## Methods

### Study Design

This study (JapicCTI‐194837) was conducted between July 8, 2019 and September 4, 2019 at Souseikai Hakata Clinic in Japan, in accordance with the International Conference on Harmonisation Good Clinical Practice guidelines, all applicable participant privacy requirements and the ethical principles outlined in the current version of the Declaration of Helsinki. The study protocol and informed consent documents were approved by the Hakata Clinic Institutional Review Board (approval number 1464BE‐7, approval date June 22, 2019). Written informed consent was obtained from each participant before the initiation of any screening evaluations.

**Figure 1:**
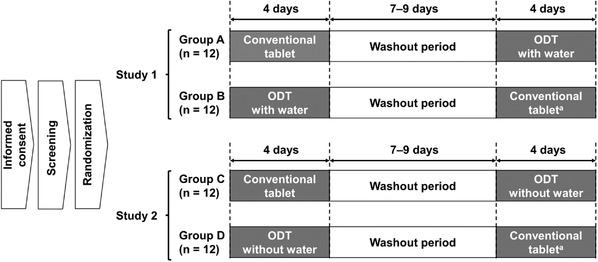
Study design. For both the esaxerenone ODT and conventional tablet, the dosage administered was 5 mg on the first day of the treatment period. All conventional tablets were taken with water. ^a^One subject each in groups B and D withdrew from the study; no subjects withdrew from groups A and C. ODT, orally disintegrating tablet.

The design was a single‐center, open‐label, randomized, two‐drug, two‐stage crossover, single‐dose study in two parts; a schematic is shown in Figure 1. Study 1 compared the bioavailability of esaxerenone ODT and conventional tablets when both were taken with water, and study 2 compared the bioavailability of the two formulations when the ODT was taken without water.

In each study, 24 subjects were randomly assigned in a 1:1 ratio to two equal groups (ie, 12 subjects per group). Groups received either the esaxerenone 5‐mg conventional tablet or esaxerenone 5‐mg ODT, followed by a washout period, and then received the alternative formulation. Subjects were required to remain at the clinic for 3 nights/4 days, from the day before administration of study treatment to 2 days after the dose was given. In both studies, treatment was administered with the subjects seated (and remaining seated for 5 hours after administration) and in the fasted state (10 hours prior to administration to 5 hours after the dose was given). Beverage consumption was prohibited from 1 hour prior to administration to 2 hours after the dose was given, with the exception of water at the time of administration. Water‐free administration of ODT required the tablet to be dissolved on the tongue using saliva; for the administration of ODT with water and conventional tablets, 100 mL of water was provided. Treatment administration compliance was assessed by a thorough oral cavity examination by the investigators.

During the hospitalization period, only water and noncaffeinated tea were allowed. Alcohol and grapefruit juice were prohibited during the entire study period, as was smoking. Standardized meals were served at appropriate times throughout the study. For 28 days before day −1 of the first treatment period until study completion, the use of other drugs or supplements was prohibited.

### Participants

The key inclusion criteria were Japanese men, aged 20–45 years, body mass index of 18.5 to <25 kg/m^2^, sitting systolic/diastolic blood pressure <140/90 mm Hg, resting pulse <99 beats per minute, and good general health as determined by medical history, clinical examination, laboratory tests, and 12‐lead electrocardiogram (ECG).

The exclusion criteria were identical to those used in a prior PK and bioavailability study of esaxerenone.^31^ In brief, subjects were excluded if they had a history of any serious disease, drug or alcohol dependence, blood‐borne infection, recent collection of whole blood or blood products, prior participation in another clinical study within 120 days before screening or any prior study involving esaxerenone, use or planned use of concomitant therapies or supplements, any abnormal clinical or laboratory findings, or any potential difficulty in attending the clinic.

### PK Sample Collection and Bioanalysis

Blood samples for the determination of esaxerenone concentration in plasma were collected before dosing and at the following nominal times (relative to the time of esaxerenone ODT or conventional tablet administration): 0.5, 1, 1.5, 2, 2.5, 3, 3.5, 4, 4.5, 5, 6, 8, 24, 48, and 96 hours. Plasma concentration was measured using a validated liquid chromatography/mass spectrometry method as published elsewhere.^9^ Briefly, esaxerenone and its internal standard (d7‐form) were extracted from plasma samples using a 96‐well solid‐phase extraction plate coated with 250 μL each of acetonitrile and water. After washing with 500 μL of acetonitrile/water (10:90, v/v), the plate was eluted with 200 μL of a diluent (acetonitrile/water 50:50, v/v). A 20‐μL aliquot was then injected into the liquid chromatograph–tandem mass spectrometer. A CAPCELL PAK C18 MGIII (Shiseido Co., Ltd., Tokyo, Japan) column (2.0 × 150 mm, 5 μm) and acetonitrile/ultrapure water (59:41, v/v) were used for chromatographic separation, and a SciexAPI 4000 (AB SCIEX, Framingham, Massachusetts) tandem mass spectrometer with TurboIonSpray source was used for detection, with electrospray ionization in the negative ion mode and multiple‐reaction monitoring of esaxerenone (*m*/*z* 465–365). The within‐ and inter‐day assay precision for quality control samples of esaxerenone was 3.7% and 4.2% at 0.3 ng/mL, 3.7% and 2.9% at 4 ng/mL, and 3.5% and 2.8% at 80 ng/mL; the assay accuracy was in the range of −1.0% to 0.3% and −0.5% to 4.3%, respectively. The lower limit of quantification was 0.1 ng/mL.

### PK Analysis

The primary outcome was the evaluation of bioequivalence of the ODT and conventional tablet, using the PK parameters maximum plasma concentration (C_max_) and AUC to the last quantifiable time (AUC_last_). Secondary outcomes included additional PK parameters: AUC_∞_, apparent total body clearance (CL/F), mean residence terminal elimination half‐life (t_1/2_), and time to reach maximum plasma concentration (T_max_). A standard noncompartmental methodology was used for the PK analyses and was calculated using Phoenix WinNonlin (Certara USA, Inc., Princeton, New Jersey).

### Safety Analysis

Safety was evaluated using reports of adverse events (AEs) and physical and laboratory testing results. AEs were categorized using the Japanese translation of the Medical Dictionary for Regulatory Activities, version 22.1. Physical and laboratory tests included hematology and urinalysis, vital signs, body weight, clinical measurements, and 12‐lead ECG.

For the safety evaluation, AEs were recorded for all subjects who provided informed consent and were enrolled in the study, regardless of whether they received treatment. All AEs were listed by event, frequency, and causal relationship to the study drug. AEs occurring during the period after the administration of the first‐phase investigational drug and prior to the administration of the second‐phase investigational drug were defined as being associated with the first‐phase formulation. AEs were considered to be associated with the second‐phase formulation if they occurred during the period after the administration of the second‐phase investigational drug up to the end of the study. For other safety parameters, frequency tables or shift tables were prepared for categorical data, and summary statistics were calculated for quantitative data.

### Sample Size Calculation and Statistical Analysis

Assuming that the ratios of C_max_ and AUC_last_ for the two formulations differ by up to 5% and taking into account PK data from a prior esaxerenone bioavailability study,^20^ the intra‐individual variations were assumed to be 12%–18% and 7%–10%, respectively. Using a standard bioequivalence ratio of 0.80–1.25, 21 subjects in each study were required to ensure the probability of the 2‐sided 90% confidence intervals (90%CIs) for the ratio of geometric least‐squares (LS) mean bioequivalence parameters in each study would be 90% or greater. Thus, in consideration of potential screening failures and withdrawal by subjects, it was decided that it was necessary to enroll a total of 48 subjects (24 per study).

The ratio (90%CI) of the geometric LS mean value of C_max_ and AUC_last_ for the two formulations was calculated for each study. All below the limit of quantification values were entered as 0 and were included as such in the calculation of means. If the 90%CIs were within the range of 0.80–1.25, the formulations were considered to be bioequivalent.

All statistical calculations were performed using SAS version 9.4 (SAS Institute, Inc., Cary, North Carolina).

## Results

### Participant Disposition and Baseline Characteristics

Overall, consent for study participation was obtained from a total of 86 subjects. Of these, 48 subjects were included in the two studies. Of the remainder, 16 subjects of 38 failed to meet the inclusion/exclusion criteria, eight of 38 withdrew consent, and 14 of 38 were released from the study following the closure of enrollment by the sponsor.

In study 1, 24 subjects judged to be eligible at the admission examination the day before the first administration of the study drug were randomly assigned to treatment. All 24 were included in the safety analyses. Of these, 23 subjects (12 in group A and 11 in group B) completed the study and were included in the PK analyses. One subject in group B discontinued the study after receiving the first dose of the study drug. The reason for discontinuation was that the subject developed an AE (dental caries), the treatment for which included anesthesia. This violated the exclusion criteria at the time of the admission examination for the second stage.

In study 2, 24 subjects judged to be eligible at the admission examination the day before the first administration of the study drug were randomly assigned to treatment. All 24 were included in the safety analyses. Of these, 23 subjects (12 in group C and 11 in group D) completed the study and were included in the PK analyses. One subject in group D discontinued the study, at his own request, after the first administration of the study drug.

Baseline characteristics of the 48 participants are shown in Table 1. There were no notable differences between treatment groups in either study. In study 1, the mean age was 23.5 years and the mean body weight was 59.0 kg. In study 2, the mean age was 25.4 years and the mean body weight was 61.9 kg.

**Table 1:** Participant Characteristics (Safety Population)

	Study 1			Study 2		
	Group A n = 12	Group B n = 12	All N = 24	Group C n = 12	Group D n = 12	All N = 24
Age (years)	24.3 ± 6.5	22.8 ± 3.8	23.5 ± 5.3	25.7 ± 6.4	25.1 ± 5.6	25.4 ± 5.9
Height (cm)	170.5 ± 4.9	170.5 ± 5.6	170.5 ± 5.1	168.7 ± 7.2	172.3 ± 5.1	170.5 ± 6.4
Body weight (kg)	58.8 ± 7.1	59.2 ± 5.2	59.0 ± 6.1	59.9 ± 5.7	63.9 ± 8.6	61.9 ± 7.4
BMI (kg/m^2^)	20.2 ± 1.7	20.3 ± 1.1	20.2 ± 1.4	21.0 ± 1.3	21.6 ± 2.0	21.3 ± 1.7

BMI, body mass index; ODT, orally disintegrating tablet.

Data are mean ± standard deviation. Group A: esaxerenone conventional tablet followed by ODT with water. Group B: esaxerenone ODT with water followed by conventional tablet. Group C: esaxerenone conventional tablet followed by ODT without water. Group D: esaxerenone ODT without water followed by conventional tablet.

### Study 1: Administration of Esaxerenone ODT With Water

**Figure 2:**
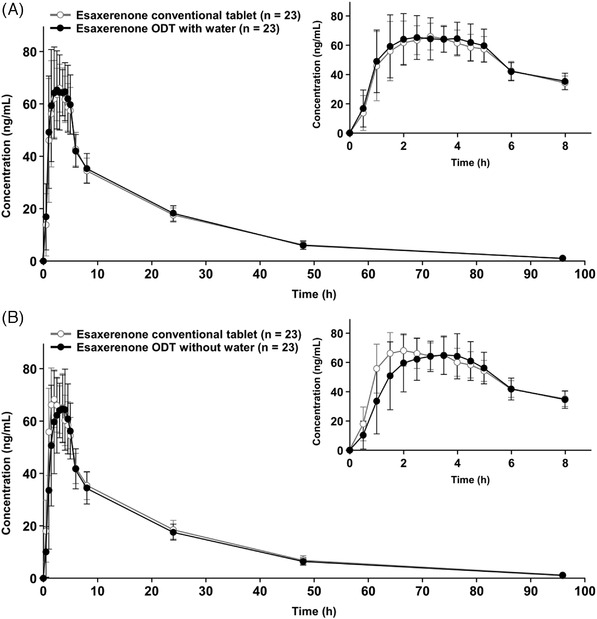
Mean ± standard deviation plasma concentration–time profiles for esaxerenone ODT and conventional tablet (pharmacokinetic population). (A) Study 1 (ODT with water). (B) Study 2 (ODT without water). The inset enlargement provides a more detailed view of the 0–8 h time period. All conventional tablets were taken with water. ODT, orally disintegrating tablet.

The mean plasma concentration–time profiles for esaxerenone ODT with water and the conventional tablet are shown in Figure 2A, and PK parameters for each formulation are shown in Table 2. The mean C_max_ values in plasma were 77.7 ng/mL (ODT with water) and 75.0 ng/mL (conventional tablet). The mean AUC_last_ values in plasma were 1290 ng·h/mL (ODT with water) and 1250 ng·h/mL (conventional tablet). The median (range) T_max_ was 2.50 hours (1.00–5.00 hours) in subjects taking ODT with water and 2.50 hours (1.00–4.50 hours) in subjects taking conventional tablets with water. Thus, the absorption rate of the conventional tablet and ODT was similarly fast, with no significant difference in absorption between the conventional tablet and ODT. The mean t_1/2_ values were 16.9 hours (ODT with water) and 16.7 hours (conventional tablet) (Table 2). There was almost no difference between the conventional tablet and ODT in the elimination rate of esaxerenone from plasma after reaching C_max_.

**Table 2:** PK Parameters for the ODT and Conventional Tablet Formulations of Esaxerenone (PK Population)

	ODT	Conventional Tablet
**Study 1: ODT with water**	N = 23	N = 23
C_max_ (ng/mL)	77.7 ± 10.3	75.0 ± 8.18
AUC_last_ (ng·h/mL)	1290 ± 180	1250 ± 152
T_max_ (h)	2.50 (1.00–5.00)	2.50 (1.00–4.50)
AUC_∞_ (ng·h/mL)	1310 ± 186	1270 ± 161
t_1/2_ (h)	16.9 ± 2.06	16.7 ± 1.89
CL/F (L/h)	3.89 ± 0.605	4.00 ± 0.511
**Study 2: ODT without water**	N = 23	N = 23
C_max_ (ng/mL)	77.0 ± 11.1	76.2 ± 10.0
AUC_last_ (ng·h/mL)	1260 ± 172	1320 ± 187
T_max_ (h)	2.50 (1.50–4.00)	2.00 (1.00–4.00)
AUC_∞_ (ng·h/mL)	1290 ± 184	1350 ± 195
t_1/2_ (h)	18.0 ± 2.42	17.5 ± 1.83
CL/F (L/h)	3.94 ± 0.566	3.79 ± 0.592

AUC_∞_, area under the plasma concentration–time curve to infinity; AUC_last_, area under the plasma concentration–time curve to the last quantifiable time; CL/F, apparent total body clearance; C_max_, maximum plasma concentration; ODT, orally disintegrating tablet; PK, pharmacokinetic; t_1/2_, terminal elimination half‐life; T_max_, time to reach maximum plasma concentration.

Data are mean ± standard deviation, except for T_max_, which is median (range).

The results of the analysis of variance for the bioequivalence evaluation are reported in Table 3. The ratios of the geometric LS mean (2‐sided 90%CIs) for ODT with water to the conventional tablet were 1.033 (0.995–1.071) for C_max_ and 1.031 (0.997–1.066) for AUC_last_; both were within the predefined range for bioequivalence (0.80–1.25).

**Table 3:** Analysis of Variance for Esaxerenone Bioequivalence Parameters (Pharmacokinetic Population)

	Geometric LS Mean		Geometric LS Mean Ratio of ODT to Conventional Tablet (90%CI)	Intrasubject %CV
	ODT	Conventional Tablet		
**Study 1: ODT with water**				
C_max_ (ng/mL)	77.050	74.621	1.033 (0.995, 1.071)	7.3
AUC_last_ (ng·h/mL)	1275.252	1237.007	1.031 (0.997, 1.066)	6.6
**Study 2: ODT without water**				
C_max_ (ng/mL)	76.226	75.430	1.011 (0.960, 1.063)	10.1
AUC_last_ (ng·h/mL)	1249.116	1305.304	0.957 (0.936, 0.978)	4.3

AUC_last_, area under the plasma concentration–time curve to the last quantifiable time; CI, confidence interval; C_max_, maximum plasma concentration; CV, coefficient of variation; LS, least squares; ODT, orally disintegrating tablet.

### Study 2: Administration of Esaxerenone ODT Without Water

The mean plasma concentration–time profiles for esaxerenone ODT without water and the conventional tablet are shown in Figure 2B, and PK parameters for each formulation are shown in Table 2. The mean C_max_ values in plasma were 77.0 ng/mL (ODT without water) and 76.2 ng/mL (conventional tablet). The mean AUC_last_ values in plasma were 1260 ng·h/mL (ODT without water) and 1320 ng·h/mL (conventional tablet). The median (range) T_max_ was 2.50 hours (1.50–4.00 hours) in subjects taking ODT without water and 2.00 hours (1.00–4.00 hours) in subjects taking conventional tablets with water. Thus, the absorption rate of the conventional tablet and ODT was similarly fast, with no significant difference in absorption between the conventional tablet and ODT. The mean t_1/2_ values were 18.0 hours (ODT without water) and 17.5 hours (conventional tablet) (Table 2). There was almost no difference between the conventional tablet and ODT in the elimination rate of esaxerenone from plasma after reaching C_max_.

The results of the analysis of variance for the bioequivalence evaluation are reported in Table 3. The ratios of the geometric LS mean (2‐sided 90%CIs) for ODT without water to the conventional tablet were 1.011 (0.960–1.063) for C_max_ and 0.957 (0.936–0.978) for AUC_last_; both were within the predefined range for bioequivalence (0.80–1.25).

### Safety

AEs are summarized in Table 4. In study 1, one subject reported a treatment‐emergent AE (TEAE) of dental caries during the administration of ODT with water, and one subject reported a TEAE of presyncope during the administration of the conventional tablet. In study 2, one subject reported a TEAE of alanine aminotransferase increased during the administration of ODT without water. All of these TEAEs were mild in severity, and none were judged by the investigator to be related to study treatment. Besides dental caries requiring treatment under anesthesia in one subject who discontinued the study in group B, all other TEAEs resolved without specific treatment. No serious or severe AEs or deaths occurred during either study. There were no obvious differences between the two formulations in any physical or laboratory test values, and no bias was observed between groups.

**Table 4:** Summary of Safety Results for the Esaxerenone Formulations (Safety Population)

	ODT	Conventional Tablet
**Study 1: ODT with water**	N = 24	N = 23
At least 1 TEAE	1 (4.2)	1 (4.3)
Treatment‐related TEAE	0	0
Serious TEAE	0	0
Discontinuation due to TEAE	0	0
Death	0	0
Description of TEAEs		
Dental caries (mild)	1 (4.2)	0
Presyncope (mild)	0	1 (4.3)
**Study 2: ODT without water**	N = 24	N = 23
At least 1 TEAE	1 (4.2)	0
Treatment‐related TEAE	0	0
Serious TEAE	0	0
Discontinuation due to TEAE	0	0
Death	0	0
Description of TEAE		
Alanine aminotransferase increased (mild)	1 (4.2)	0

ODT, orally disintegrating tablet; TEAE, treatment‐emergent adverse event.

Data are n (%). TEAEs were categorized using the Japanese translation of the Medical Dictionary for Regulatory Activities, version 22.1.

## Discussion

Hypertension is an important clinical issue in Japan, and the provision of adequate treatment for patients who are unable to take conventional oral tablets is key to improving treatment adherence and blood pressure control. Esaxerenone has proven efficacy in the conventional tablet form,^12^, ^13^, ^14^, ^15^, ^16^, ^17^, ^18^, ^19^ and this study demonstrated that the 5‐mg ODT formulation taken with or without water is bioequivalent to the 5‐mg conventional esaxerenone tablet as the 90%CIs fell within the predefined bioequivalence range of 0.80–1.25.

The mean ± standard deviation parameters of C_max_ and AUC_last_ observed in this study with the 5‐mg conventional tablet were consistent with the values previously reported in healthy adults (64.9 ± 12.1 ng/mL and 1200 ± 174 ng h/mL, respectively).^31^ This supports the consistency of the reference data in this study and signifies that the bioequivalence analysis is robust, suggesting that esaxerenone ODT will be equally effective as the conventional tablet in patients with hypertension.

Notably, the C_max_ and T_max_ values of esaxerenone ODT without water were almost the same as those of the conventional tablet. For C_max_, results were comparable with those of a previous clinical exploration of esaxerenone PK,^31^ and the values were within the previously established range of variability. Values of T_max_ were also within the range of variability established for the 5‐mg conventional tablet.^31^ In addition, the AUC and t_1/2_ values observed in our study were similar between ODT without water and the conventional tablet. There was no difference in systemic exposure (AUC and C_max_) between with and without water conditions for ODT. In the context of a drug that is designed to be administered daily for an extended duration in patients with hypertension, minor within‐range variability in C_max_ and T_max_ between formulations is not considered to be clinically meaningful, particularly as the overall bioequivalence of the formulations was confirmed.

There was no clear difference in the incidence of TEAEs between the two formulations, and no discontinuations due to AEs with either formulation. Of the TEAEs that were reported, none were considered by the investigator to be related to study treatment, and all were mild. Overall, we consider that the ODT formulation of esaxerenone has a safety profile similar to that of the conventional oral tablet.

The main limitation of our study is that it included healthy male subjects and had a single‐center, open‐label, single‐dose design. Therefore, there was a limited assessment of dosing convenience.

## Conclusion

This study confirmed that esaxerenone 5‐mg ODT taken with or without water was bioequivalent to the 5‐mg conventional oral tablet. As the ODT formulation may help achieve the correct peroral administration of esaxerenone, particularly in populations where difficulty swallowing or inability to swallow solid tablets may hinder compliance, clinicians may wish to consider replacing the conventional tablet formulation with the ODT.

## Conflicts of Interest

AK, JT, YI, YN, and HI are employees of Daiichi Sankyo Co., Ltd. AK, JT, YI, and HI are shareholders of Daiichi Sankyo Co., Ltd. TE has no potential conflicts of interest to disclose.

## Funding

This study was funded by Daiichi Sankyo Co., Ltd., Tokyo, Japan.

## Author Contributions

AK, TE, JT, YI, YN, and HI were responsible for the study design and conduct, and data analysis and interpretation. All authors were involved in writing and reviewing the manuscript, and all authors provided final approval of the manuscript for submission.

## Data Sharing

All de‐identified patient data relevant to this study are included in this article. Additional data and supporting documents pertaining to this study are provided upon reasonable request made via this web address (https://vivli.org/ourmember/daiichi-sankyo/) in accordance with the data sharing policy of Daiichi Sankyo Co., Ltd.
